# Staple Line Erosion in a Neobladder Causing Postoperative Hematuria

**DOI:** 10.7759/cureus.15450

**Published:** 2021-06-04

**Authors:** Danyon J Anderson, Matthew Kasson, Mit Patel, Nathan Li, Peter Langenstroer

**Affiliations:** 1 School of Medicine, Medical College of Wisconsin, Wauwatosa, USA; 2 Urology, Medical College of Wisconsin, Wauwatosa, USA

**Keywords:** surgical staple, neobladder, staple line erosion, urothelial malignancy, cystoprostatectomy

## Abstract

Erosion of metal surgical staples is a rare but described surgical complication. Staple erosion in a bladder may present with hematuria, urinary tract infection (UTI), bladder pain, or fistula. We present a 64-year-old male with a history of urothelial carcinoma treated by cystoprostatectomy with neobladder reconstruction. Over the next two to three years, he developed hematuria and had multiple urine cytologies suspicious for cancer. The cystoscopic evaluation revealed staple line erosion but no cancer. We believe this to be the first published case of symptomatic staple erosion in a neobladder. Clinicians should be aware that staple line erosion in a neobladder can occur and mimic malignancy recurrence.

## Introduction

Erosion of metal surgical staples, like those used in this case, is a rare but described complication of surgery. There have been reported cases of staple erosion after a myriad of procedures including neo-esophageal creation, colposuspension, and oophorectomy [1-6]. Reported effects of staple erosion into the bladder include bladder pain, hematuria, urinary tract infection, and fistula [1-4]. The effect of staple erosion on lab urine cytologies for urothelial carcinoma has not been studied. To our knowledge, no cases of staple line erosion in a neobladder have been reported. We present a case of staples eroding into a patient’s bladder after cystectomy and neobladder creation, leading to subsequent hematuria and urine cytologies suspicious for urothelial carcinoma recurrence. Presumably, this is the first case of metal staple erosion in a neobladder.

## Case presentation

**Figure 1:**
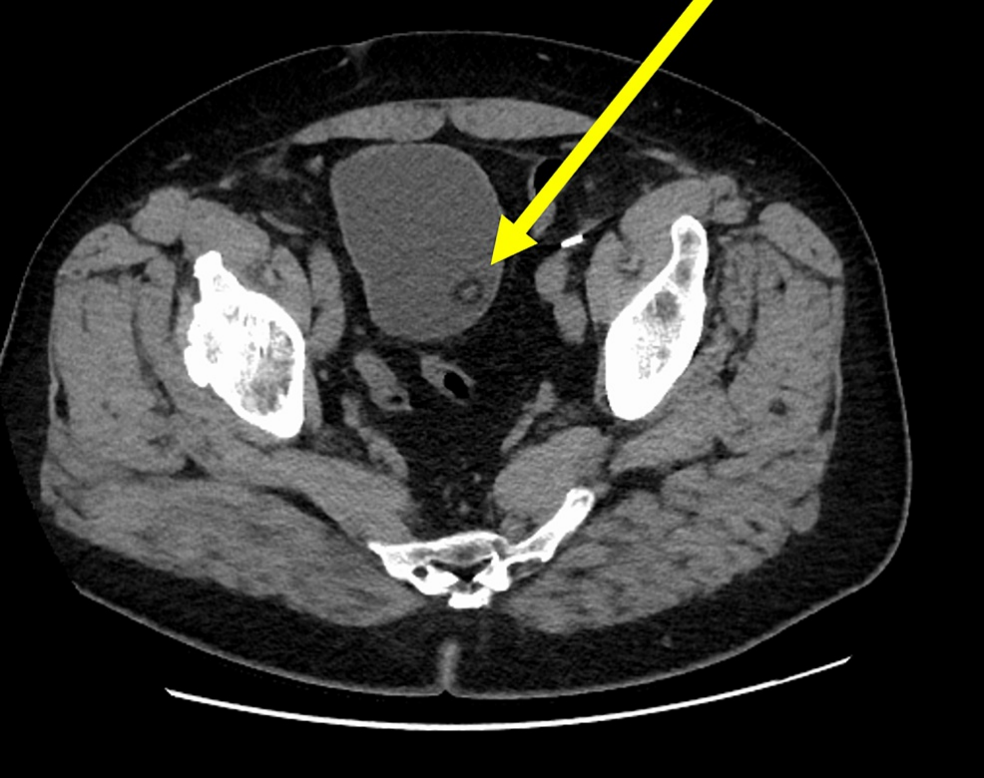
CT Urogram Showing Calcification of Neobladder From Staple Line Erosion

**Figure 2:**
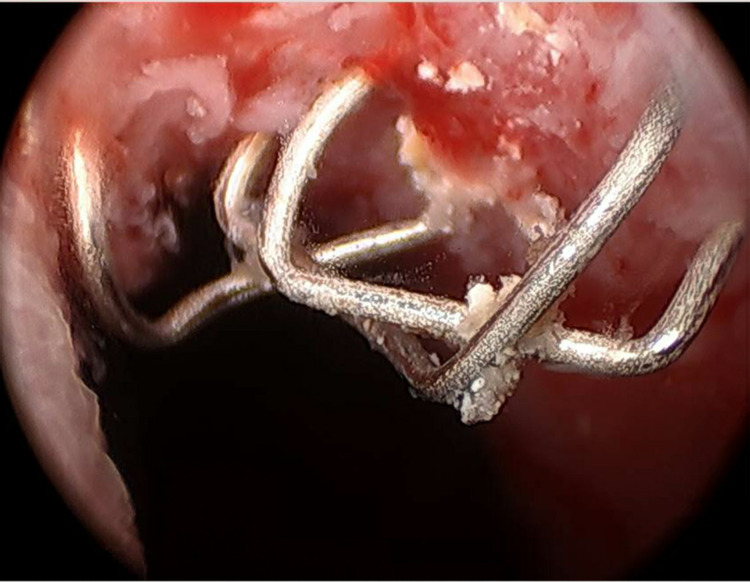
Cystoscopic Picture of Staple Line Erosion Into Neobladder

A 64-year-old male presented to the clinic six years ago with hematuria and was diagnosed with urothelial carcinoma in situ. The patient subsequently underwent two cycles of Bacillus Calmette-Guérin (BCG) bladder instillation, valrubicin instillation, and mitomycin instillation with persistent biopsy-proven disease recurrence resulting in him undergoing cystoprostatectomy with Studer neobladder reconstruction. The patient’s disease resection was staged as pT3aN0M0 with negative margins. The patient followed the typical postoperative course and was later discharged. Two years following the patient’s procedure, he presented to the clinic for multiple suspicious urine cytologies. The subsequent ureteroscopic investigation was notable for a suspicious left-sided renal lesion, and the patient was started on antegrade BCG instillation. The lesion regressed and was no longer identifiable. The patient did well until one year later, at which time he reported persistent gross hematuria with normal hemoglobin of 14.1 g/dL. Subsequent flexible cystoscopy and CT urogram were notable for several neobladder wall calcifications at the 12 o'clock position at the bladder neck (Figure 1). The patient underwent rigid cystoscopy, at which time the source of this abnormality was found to be staples eroding through the neobladder wall (Figure 2). No other suspicious lesions were identified by cystoscopy or CT urogram. The staples were noted to be quite encrusted; however, the staples were able to be subsequently grasped and removed using endoscopic biopsy forceps. Adequate hemostasis was achieved without the use of cautery.

## Discussion

This is the first reported case of metal staple erosion into a neobladder and the first association of eroded staples with urine cytologies suspicious for urothelial carcinoma. Since this study is the first report of such a staple erosion, it is difficult to draw conclusions regarding the prevalence of staple erosion following cystoprostatectomy with bladder reconstruction and the connection between erosion and positive cancer cytology.

However, this case still informs practice in two ways. First, it is possible for metal staples to erode following the construction of a neobladder. Signs and symptoms of staple erosion may include bladder pain, hematuria, bladder wall calcifications, and urine cytologies suspicious for urothelial cell carcinoma. These are consistent with previously reported symptoms of metal staple erosion [1-4]. Second, although non-visualized recurrence of carcinoma in situ and other previously established etiologies may be more likely, staple erosion may be considered a possible cause for positive urine cancer cytology. The specific positive cytology was aneuploidy of chromosomes 3, 7, and 17 detected on fluorescence in situ hybridization (FISH). The specificity of urine FISH for urothelial carcinoma is 45% in patients aged 60-70 [7]. Inflammation is a common cause of false-positive urine FISH for urothelial carcinoma [8]. Staple erosion causes inflammation; thus, staple erosion may lead to a false-positive urine FISH for urothelial cell carcinoma.

Metal staples can be a cause of hematuria (due to erosion) in a neobladder. We encourage further publishing of reports of staple erosion into neobladders.

## Conclusions

We report what we believe to be the first case of metal staple erosion into a neobladder following cystoprostatectomy with bladder reconstruction. The patient’s postoperative course was further complicated by urine cytologies suspicious for urothelial carcinoma, with new onset of gross hematuria raising concern for disease recurrence. In the absence of alternative etiologies, staple erosion should be considered in patients with hematuria following neobladder creation.

## References

[1] Chen YY, Chang JM, Lai WW (2012). Tracheo-neo-esophageal fistula caused by exposed metallic staples erosion. Ann Thorac Surg.

[2] Washington JL (2002). Staple erosion into the bladder after mesh and staple laparoscopic colposuspension. A case report. J Reprod Med.

[3] Rettenmaier CR, Abaid LN, Hu JC, Brown JV 3rd, Micha JP, Goldstein BH (2009). Delayed staple erosion into the bladder after removal of a benign ovarian mass. J Minim Invasive Gynecol.

[4] Kaye GL, McCormick PA, Siringo S, Hobbs KE, McIntyre N, Burroughs AK (1992). Bleeding from staple line erosion after esophageal transection: effect of omeprazole. Hepatology.

[5] Kaye GL, McCormick PA, Siringo S, Hobbs KE, McIntyre N, Burroughs AK (1991). Staple-line erosion: a common source of recurrent bleeding following stapled oesophageal transection. Br J Surg.

[6] Al-Khudari S, Succar E, Standring R, Khadra H, Ghanem T, Gardner GM (2013). Delayed failure after endoscopic staple repair of an anterior spine surgery related pharyngeal diverticulum. Case Rep Med.

[7] Gopalakrishna A, Longo TA, Fantony JJ, Owusu R, Foo WC, Dash R, Inman BA (2016). The diagnostic accuracy of urine-based tests for bladder cancer varies greatly by patient. BMC Urol.

[8] Todenhöfer T, Hennenlotter J, Kühs U (2012). Influence of urinary tract instrumentation and inflammation on the performance of urine markers for the detection of bladder cancer. Urology.

